# Comment on: Macronutrient Intake and Risk of Crohn’s Disease: Systematic Review and Dose–Response: Meta-Analysis of Epidemiological Studies, *Nutrients* 2017, *9*, 500

**DOI:** 10.3390/nu9090932

**Published:** 2017-08-24

**Authors:** Yong-Fang Zhang, Zheng-Ke Xiang, Chang-Zhao Liu

**Affiliations:** 1 Department of Pediatrics, The Central Hospital of Enshi Autonomous Prefecture, Enshi 445000, China; gtzqy@163.com (Y.-F.Z.); xiangzhengke22@163.com (Z.-K.X.); 2 Department of Cardiology, The Central Hospital of Enshi Autonomous Prefecture, Enshi 445000, China

We read with great interest the article by Zeng et al. recently published in *Nutrients* [1]. Without doubt, their work was well-designed and is the first dose–response meta-analysis focusing on macronutrient intake and risk of Crohn’s disease (CD).

The authors suggested a lack of association between total carbohydrate, fat, or protein intake and CD risk, which contradicts current conventional views. During the past decades, the increasing prevalence of western diet coincides with an increasing incidence of CD in those regions with a primary low incidence [2]. Thus, the western diet is usually regarded as a risk factor for CD. In validation, the western diet can induce small intestinal epithelial dysfunction by affecting toll-like receptors in mice [3]. Importantly, the western diet is characterized by high quantities of fat, sugar, and animal protein, and low consumption of fruits and vegetables [4]. As fruit and vegetable consumption is inversely associated with CD risk [5], it is easy to speculate a potential association between high intake of fat, sugar, and animal protein and CD risk. In animal models, both high-fat diet and high-protein diet can exacerbate experimental colitis, which is consistent with the speculation [6,7]. Furthermore, the authors found no subtypes of unsaturated fat in association with CD risk, although some proved anti-inflammatory and effective in CD treatment [8].

We thought the inconsistency with animal studies might contribute to the complex etiology of CD. For example, both genetic and environmental factors play a critical role in the development of CD [9]. However, most epidemiological studies ignore the effect of genetic susceptibility, and the results are only adjusted by environmental factors like drinking and smoking. In the Costea et al. study, children with a higher dietary ratio of n6/n3 polyunsaturated fatty acids (PUFA) were susceptible to CD if they were also carriers of specific variants of *CYP4F3* and *FADS2* genes [10]. This indicates a diet–gene interaction in CD pathogenesis. Dietary nutrients might also play a significant role through interacting with other factors.

In summary, the role of macronutrient intake is controversial in CD pathogenesis, and large-scale prospective studies considering more confounders are needed to clarify the role.
